# Add-On Treatment with Gliclazide for Cancer Patients with Type 2 Diabetes Undergoing Cyclic Glucocorticoid-Containing Chemotherapy

**DOI:** 10.3390/biomedicines13051101

**Published:** 2025-05-01

**Authors:** Seung Eun Lee, Ju-Hyun Park, Dalyong Kim, Hyun-A Lee, Yun Seong Kang, Young Soon Yoon, Yun Jeong Jeong, Han Seok Choi, Kyoung-Ah Kim

**Affiliations:** 1 Internal Medicine, Dongguk University Ilsan Hospital, Goyang 10326, Republic of Korea; seyi0918@naver.com (S.E.L.); moondragon81@naver.com (D.K.); rk0496@naver.com (H.-A.L.); oak118@hanmail.net (Y.S.K.); ysyoonmd@naver.com (Y.S.Y.); yunjj.tomato@gmail.com (Y.J.J.); hschoi402@gmail.com (H.S.C.); 2 Department of Statistics, Dongguk University, Seoul 04620, Republic of Korea; juhyunp@dongguk.edu

**Keywords:** diabetes, cancer, chemotherapy, dexamethasone, continuous glucose monitoring, hyperglycemia, glucocorticoid

## Abstract

**Aims**: Despite its high prevalence, studies on glucocorticoid-induced hyperglycemia are lacking. We examined the glucose profiles of patients with type 2 diabetes undergoing dexamethasone-containing chemotherapy using continuous glucose monitoring (CGM). We also investigated the effects of gliclazide on the management of hyperglycemia in these patients. **Materials and Methods**: Seventeen patients with type 2 diabetes who received cyclic chemotherapy with dexamethasone were enrolled in this study. During the first cycle, iPro2, a blinded CGM device, was applied for 7 days. If a patient’s CGM data exhibited an increase of 20% or more in the mean glucose level after dexamethasone administration, they received the second cycle, unless they had already received sulfonylurea or their chemotherapy regimen had changed. During the second cycle, the patients were treated with gliclazide as an add-on to their routine diabetic medication. **Results**: Dexamethasone treatment significantly increased glucose levels, especially in patients with a longer diabetes duration (8.4 years vs. 1.2 years, *p* = 0.009). For the nine patients who proceeded to the second cycle, gliclazide treatment significantly ameliorated hyperglycemia. Time in range increased from 33.11% to 45.22% (*p* = 0.020), and time above range significantly decreased from 66.89% to 52.78% (*p* = 0.003). The glucose management indicators were 9.52% and 8.40% for pre- and post-gliclazide treatment, respectively. One patient visited the emergency department because of symptomatic hypoglycemia. **Conclusions**: Chemotherapy regimens containing dexamethasone result in high blood glucose levels even after the last dexamethasone dose in patients with pre-existing diabetes. Adding gliclazide could be beneficial in managing hyperglycemia during dexamethasone-containing chemotherapy.

## 1. Introduction

Type 2 diabetes and cancer are two prevalent medical conditions worldwide with increasing incidence [1]. Patients with cancer and concomitant diabetes exhibit decreased physical performance and low vitality [2]. These patients also have higher mortality than cancer patients without diabetes [3]. Furthermore, cancer patients who have uncontrolled hyperglycemia have a higher risk of poor survival than those with well-controlled diabetes [4].

Patients undergoing cyclic chemotherapy face a heightened risk of hyperglycemia, primarily due to the use of glucocorticoids, which are used as antiemetics or to prevent allergies. Although the incidence of glucocorticoid-induced hyperglycemia varies based on the dosage and duration of glucocorticoid use, a meta-analysis reported the incidence of hyperglycemia in approximately 32% of patients without diabetes [5]. In another study, all patients with pre-existing diabetes experienced hyperglycemia following glucocorticoid treatment [6].

Despite its high prevalence, there is a paucity of research on glucocorticoid-induced hyperglycemia, hindering the development of a comprehensive guideline. Recently published studies have proposed management strategies for glucocorticoid-induced hyperglycemia, indicating that insulin therapy is often inevitable [7,8]. However, initiating insulin in cancer patients undergoing chemotherapy poses challenges due to their frailty and the burden of cancer itself, in addition to the fear of self-injecting insulin. Additionally, cyclic short-term exposure to glucocorticoids complicates matching hyperglycemic oscillations with insulin.

The increased availability of continuous glucose monitoring (CGM) in recent times has facilitated the development of a detailed understanding of glucose profiles, allowing for the capture of glycemic variability and short-term glucose patterns in glucocorticoid-induced hyperglycemia [9,10]. This capability would be particularly valuable in the management of glucocorticoid-induced hyperglycemia in patients undergoing chemotherapy exhibiting high glucose oscillations. However, only a few studies have used CGM to investigate glucocorticoid-induced hyperglycemia in patients with cancer receiving chemotherapy, irrespective of diabetes status [11,12,13,14].

Sulfonylureas enhance insulin release from pancreatic beta cells, with their immediate onset of action proving beneficial in managing short-term glucocorticoid-induced hyperglycemia. Glimepiride, a second-generation sulfonylurea, was reported to improve fasting glucose levels in glucocorticoid-induced diabetes [15]. Another second-generation sulfonylurea, gliclazide, is noted for its low risk of hypoglycemia due to its inactive metabolites [16]. The Joint British Diabetes Societies recommends the morning administration of gliclazide to manage the glucose excursion associated with once-daily oral steroid treatment [17]. However, this recommendation is consensus-based, not evidence-based, necessitating further investigation.

This study aimed to assess the effectiveness of gliclazide administration in managing glucocorticoid-induced hyperglycemia. Given the substantial glycemic fluctuations during cyclic chemotherapy, we employed a pre–post comparison across consecutive chemotherapy cycles to minimize inter-individual variability and confounding chemotherapy regimen changes. Additionally, we utilized CGM to comprehensively capture glycemic variability following glucocorticoid administration and subsequent gliclazide treatment.

## 2. Materials and Methods

### 2.1. Study Design

The study participants were individuals with type 2 diabetes, aged between 19 and 84 years, who were scheduled to receive cyclic chemotherapy including glucocorticoids at Dongguk University Ilsan Hospital and provided informed consent. We enrolled 17 patients who had undergone a median of 4 days of dexamethasone treatment between April 2020 and September 2022. The study spanned one or two consecutive cycles of chemotherapy, determined by glucose level after dexamethasone treatment (Supplementary Figure S1). Patients who exhibited significant hyperglycemia during the first cycle, did not take sulfonylurea, and did not have their chemotherapy regimen changed proceeded to the second cycle, in which they received gliclazide; otherwise, only the first cycle was conducted. Significant hyperglycemia was defined as having a 20% or greater increase in the mean glucose value post-dexamethasone administration compared to that on day 0 (D0), which refers to the day before treatment. A total of nine patients completed the second cycle. This study was performed in accordance with the Declaration of Helsinki, and written informed consent was obtained from each patient. This study was approved by the Institutional Review Board (IRB) of Dongguk University Ilsan Hospital (IRB No.: 2019-10-018).

### 2.2. Treatment with Gliclazide

Among the nine eligible patients, immediate-release gliclazide tablets (80–160 mg) were administered during the second cycle of chemotherapy on the days they received dexamethasone. On D1, gliclazide was taken at the same time as dexamethasone administration, and from D2 onwards, it was taken in the morning. Gliclazide was added to the patients’ existing antidiabetic regimens without modification or discontinuation of their baseline medications.

For individuals aged >70 years or those who had renal dysfunction (estimated glomerular filtration rate <45 mL/min/1.73 m^2^) or hepatic dysfunction (Child–Pugh class B or C), 80 mg of gliclazide was administered; otherwise, 160 mg was administered.

### 2.3. CGM and Self-Monitoring of Blood Glucose (SMBG)

During the first cycle, a blinded CGM device (iPro2, Medtronic, Northridge, CA, USA) was applied for 7 days (From D0 to D6). The choice between an inpatient or outpatient setting depended on the chemotherapy regimen and patient preference. In the inpatient setting, nurses checked capillary blood glucose levels four times daily (ACCU-CHEK^®^ Inform II, AccuCheck, Roche Diagnostics, Indianapolis, IN, USA), while in the outpatient setting, patients were instructed to measure capillary blood glucose levels using a glucometer (Accu-Chek^®^ Guide, AccuCheck, Roche Diagnostics, USA) four times daily for calibration. CGM-derived metrics, including the time in range (TIR, 70–180 mg/dL), time above range (TAR, >180 mg/dL), time below range (TBR, <70 mg/dL), glucose management indicator (GMI), and standard deviation (SD), were obtained.

### 2.4. Statistical Analysis

Descriptive statistics were used to summarize the baseline characteristics. Normally distributed variables are presented as the mean ± SD, while skewed data are expressed as the median (25 and 75% interquartile range). Statistical comparisons of baseline characteristics were performed between patients with and without hyperglycemia after dexamethasone treatment. Continuous variables were analyzed using Student’s *t*-test or a Mann–Whitney U test, depending on the normality of the data. Categorical variables were assessed using the chi-squared test or Fisher’s exact test.

To ascertain the temporal trajectory of glucose levels without assuming a specific functional form, this study employed the locally weighted scatterplot smoothing (LOWESS) method as a nonparametric regression model for the gathered dataset. Within the LOWESS framework, a quadratic regression model was specified for each localized region, encompassing 25% of the total data points, constituting the requisite model parameters. To compare the glucose area under the LOWESS curve (AUC) between two cycles, a bootstrap *p*-value [18] was calculated based on 1000 bootstrap resamples generated with replacement from the original dataset.

To quantitatively interpret the nonlinear patterns of glucose levels before and after dexamethasone treatment, linear spline regression models with three knots distributed over time were introduced to facilitate the estimation of hourly changes. This analysis was conducted separately for patients involved solely in the first treatment cycle and for those participating in both the first and second cycles. Three knots that reflected inflection points were selected based on a smoothing curve [19]. Knot 1 was at 1 h before dexamethasone treatment; knot 2 and knot 3 were at 5.5 h and 55.5 h after dexamethasone treatment, respectively. This linear spline produced four different linear slopes of glucose measure: <−1 h, −1 h to 5.5 h, 5.5 h to 55.5 h, and ≥55.5 h. In an exploratory analysis, an alternative spline model was also conducted using knots at −1 h, 5.5 h, and 80 h after dexamethasone treatment.

Furthermore, paired *t*-tests were used to compare CGM parameters before and after gliclazide treatment. The analyses were conducted using R version 4.3.1 (R Core Team, Vienna, Austria), and a *p*-value of 0.05 was considered statistically significant.

## 3. Results

Table 1 shows the baseline patient characteristics. The mean age was 63.1 years, with 11 out of 17 (64.7%) patients being male. The mean glycated hemoglobin (HbA1c) level was 7.5%, and the median fasting blood sugar level was 141 mg/dL. The mean duration of diabetes was 5.9 years. Regarding underlying malignancies, eight (47.1%), six (35.3%), and three (17.6%) patients had gastrointestinal cancer, lung cancer, and gallbladder–biliary cancer, respectively. Patients with hyperglycemia after the first cycle of dexamethasone treatment had a significantly longer duration of diabetes compared to that of those without hyperglycemia (8.4 ± 7.4 years vs. 1.2 ± 1.4 years, *p* = 0.009). Although not statistically significant, the proportion of patients receiving combination therapy tended to be higher in the hyperglycemia group compared to the non-hyperglycemia group (90.9% vs. 50%, *p* = 0.193). All patients in the dual combination group received metformin plus a dipeptidyl deptidase-4 (DPP-4) inhibitor. Among those in the triple therapy group, most combinations also included metformin and a DPP-4 inhibitor, in combination with agents such as sulfonylureas, SGLT2 inhibitors, TZDs, or insulin. Patients in the hyperglycemia group tended to have higher HbA1c levels (7.9 ± 1.7% vs. 6.8 ± 1.4%, *p* = 0.216) and fasting blood sugar levels (median [IQR]: 145.0 [131.0–170.0] vs. 126.0 [111.0–142.0] mg/dL, *p* = 0.606) compared to those in the non-hyperglycemia group.

**Figure 1:**
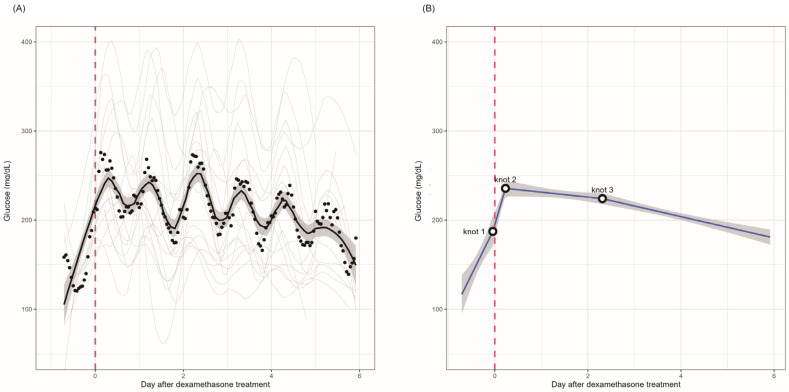
Changes in glucose levels after the first cycle of chemotherapy including dexamethasone treatment (n = 17). (**A**) Patient-specific smooth curves in gray, generated through the application of the LOWESS method for patients undergoing the first cycle of chemotherapy. The black solid line and gray-shaded area depict the temporal mean glucose trajectory and 95% pointwise confidence intervals, respectively. (**B**) A plot of the linear spline model with three knots positioned at 1 h before dexamethasone treatment, and 5.5 h and 55.5 h after dexamethasone treatment. The gray-shaded area represents the 95% pointwise confidence intervals. LOWESS, locally weighted scatterplot smoothing.

The smooth curves generated for patients in the first cycle using the LOWESS method are shown in Figure 1A, while Figure 1B shows the linear spline interpolation of blood glucose levels. During the first cycle, patients experienced an increase in mean glucose levels before dexamethasone treatment (Figure 1A). Glucose levels sharply increased after dexamethasone treatment and remained high until 2–3 days after dexamethasone treatment. Subsequently, the glucose levels steadily decreased. Linear spline interpolation indicated an estimated mean glucose change of 7.44 (from 4.36 to 10.52) mg/dL per hour from 1 h before to 5.5 h after dexamethasone treatment (Figure 1B; Table 2). Glucose levels did not change significantly from 5.5 h to 55.5 h after dexamethasone treatment (estimated mean glucose change: −0.23 [from −0.48 to 0.02] mg/dL per hour, *p* = 0.071), followed by a subsequent decline (estimated mean glucose change: −0.50 [from −0.64 to −0.35] mg/dL per hour, *p* < 0.001).

For patients who completed both the first and second cycle of CGM, the baseline characteristics are presented in Supplementary Table S1. The increase in glucose levels after dexamethasone treatment was significantly alleviated after gliclazide treatment (estimated mean glucose change before and after gliclazide treatment: 9.26 [from 5.31 to 13.22] mg/dL per hour vs. 0.82 [from −2.91 to 4.56] mg/dL per hour) (Supplementary Figure S2; Table 2). The difference between the first and second cycles was 8.44 (from 3.00 to 13.88) mg/dL per hour (*p* = 0.002). Across both cycles, glucose levels remained elevated from 5.5 h to 55.5 h after dexamethasone treatment, followed by a significant decrease (Table 2).

**Figure 2:**
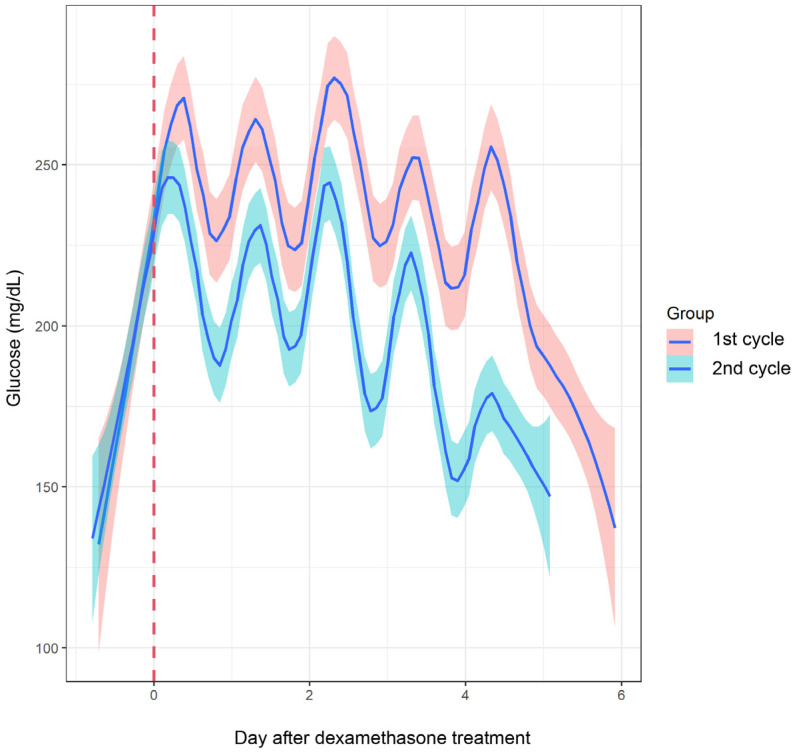
Changes in glucose levels from the first to second cycles of dexamethasone-containing chemotherapy (n = 9). The temporal trajectories of the mean glucose levels across two cycles, with 95% pointwise confidence intervals represented by the shaded area. The color scheme distinguishes between the first (depicted in pink) and second (depicted in blue) cycles of chemotherapy.

Comparing the glucose AUC values of smooth curves, the AUC from D1 to D5 after dexamethasone treatment was significantly higher in the first cycle than in the second cycle (1206.6 mg·h/dL in first cycle vs. 997.3 mg·h/dL in second cycle, *p* < 0.001) (Figure 2).

Table 3 shows the mean values of the CGM parameters of the participants before and after gliclazide treatment. Following gliclazide treatment, TIR significantly increased (33.11% vs. 45.22%, *p* = 0.020), whereas TAR significantly decreased (66.89% vs. 52.78%, *p* = 0.003). The GMI and mean glucose levels were significantly reduced after gliclazide treatment (GMI, 9.52% vs. 8.40%, *p* = 0.020; mean glucose, 226.67 mg/dL vs. 194.33 mg/dL, *p* = 0.022). There was no change in the SD values before and after gliclazide treatment.

One patient visited the emergency department on the morning of chemotherapy day 4 (1 day after the last dose of dexamethasone), presenting with hypoglycemic symptoms and an SMBG of 50 mg/dL. Upon arrival, the patient consumed a banana in the emergency department, resulting in a glucose level of 134 mg/dL. The patient was discharged without any additional intervention.

## 4. Discussion

In this study, we observed a rapid increase in glucose levels during a median of 4 days of treatment with dexamethasone-containing chemotherapy, leading to hyperglycemia that persisted for approximately 2–3 days before gradually decreasing. Treatment with gliclazide notably ameliorated hyperglycemia, as indicated by the increased TIR and decreased TAR.

Significant hyperglycemia after dexamethasone treatment was more frequently observed in patients with a longer duration of diabetes. Additionally, those with significant hyperglycemia tended to exhibit suboptimal glucose control or had to be managed with combination therapy before dexamethasone treatment. These findings align with previous reports indicating that factors such as underlying glucose intolerance, reduced sensitivity to insulin, impaired insulin secretion stimulated by glucose, or a family history of diabetes are risk factors for glucocorticoid-induced hyperglycemia [20]. Previously, a study noted the persistence of dexamethasone-induced hyperglycemia for approximately 23–35 h after a single dose of intravenous dexamethasone [11]. This duration appears longer than what was observed in our linear spline model, which indicated 55 h of hyperglycemia during a median of 4 days of dexamethasone treatment. The knot (55 h) was determined based on the inflection point of the smoothing curve. However, the curve showed the gradual amelioration of hyperglycemia, with peak glucose levels on days 4 and 5 being lower than those on days 3 and 4, respectively. The gradual improvement in hyperglycemia in our study could be attributed to the differing duration of dexamethasone usage, which ranged from 3 to 6 days. Notably, exploratory linear spline interpolation revealed that significant hyperglycemia persisted until 80 h (estimated mean glucose change: −0.63 [from −0.85 to −0.41] mg/dL per hour from 80 h after dexamethasone treatment; Supplementary Table S2).

Although treatment with gliclazide significantly improved glucocorticoid-induced hyperglycemia, the TIR was still notably lower than that recommended in the guideline [21]. Currently, there is a paucity of research investigating glycemic control in patients with cancer undergoing chemotherapy. In a study by Legris et al. [14], the TIR of patients treated with chemotherapy was reported to be 67.2%, which is remarkably higher than that in our study. However, the proportion of patients receiving glucocorticoid treatment in the study was 81.2%, and only 26.1% of them received repeated treatment with glucocorticoids, which might account for better glycemic control than that seen in our study. Conversely, Gerards et al. [12] reported that the TIR of patients treated with repeated glucocorticoid administration was 20.9–34.4% despite intensive insulin treatment with a mean of 26–40.3 IU of insulin per day. Our study aligns with these findings, highlighting challenges in managing glucocorticoid-induced hyperglycemia in cancer patients.

The difficulties in treating glucocorticoid-induced hyperglycemia in cancer patients appear to be explained not only by the effect of the glucocorticoid itself but also by several factors. Chemotherapy-related nausea, diminished oral intake, or the rapid infusion of dextrose fluid can drastically alter glucose levels. In addition, social determinants of health may make it difficult to manage hyperglycemia in cancer patients, as they are likely to experience psychological, physical, and financial burdens [22]. Implementing self-care for diabetes may be challenging. In our experience, the affordability of glycemic control varies significantly across patients, emphasizing the need for individualized treatment for hyperglycemia in cancer patients.

One patient presented at the emergency department with symptomatic hypoglycemia on chemotherapy day 4 (1 day after the last dose of dexamethasone), consistent with the results of a previous study in which all hypoglycemic episodes occurred at the end of each cycle (days 3–5) [12]. Therefore, special attention should be paid to hypoglycemia at the end of glucocorticoid administration when managing glucocorticoid-induced hyperglycemia.

The strengths of our study include its meticulous examination of glucose levels using CGM in chemotherapy-treated patients and its investigation of the effects of gliclazide, which have rarely been tested previously. However, this study had some limitations. First, the sensor wear time varied across patients, resulting in a difference in the CGM device wear time between the first and second cycles of chemotherapy. Although patients were encouraged to wear the CGM device for several days, starting 1 day before dexamethasone administration, some patients opted not to visit the hospital before the start of chemotherapy, which made a difference in CGM device wear time. Nevertheless, we compared the AUC of the first and second cycles for the same period (from D1 to D5). Second, the sample size was relatively small, which may have limited the statistical power of this study to detect significant differences. Third, changes in dietary intake and physical condition during consecutive chemotherapy cycles could have independently affected glucose levels. Ideally, a controlled study design using a placebo, or an active comparator would have better accounted for such confounding factors; however, this approach was not employed in our study. Also, although we used blinded CGM, the SMBG results might have led to behavioral modifications that resulted in an improvement in hyperglycemia, in addition to the effect of gliclazide. Future prospective studies with larger sample sizes and a controlled design are needed to confirm the findings of our study.

## 5. Conclusions

In conclusion, chemotherapy regimens containing dexamethasone result in elevated blood glucose levels, even after the last dose of dexamethasone, in patients with pre-existing diabetes. The risk of hyperglycemia is more pronounced in patients with a longer duration of diabetes and suboptimal glucose control, or those who have been treated with multiple antidiabetic medications. The addition of gliclazide could serve as a viable alternative for patients unable to receive multiple insulin injections.

## Figures and Tables

**Table 1:** Baseline characteristics of study population.

	All Patients (n = 17)	Hyperglycemia After DXM (n = 11)	No Hyperglycemia After DXM (n = 6)	*p*-Value
Age	63.1 ± 10.7	64.2 ± 10.1	61.2 ± 12.4	0.594
Male (%)	11 (64.7)	7 (63.6%)	4 (66.7%)	1.000
Height (cm)	162.5 ± 8.9	163.5 ± 9.8	160.7 ± 7.6	0.552
Weight (kg)	61.3 ± 9.8	62.3 ± 11.0	59.5 ± 7.8	0.584
BMI (kg/m^2^)	23.2 ± 2.9	23.3 ± 3.4	23.0 ± 1.6	0.813
Waist circumference (cm)	85.4 ± 8.0	83.6 ± 8.2	88.8 ± 6.8	0.212
Cancer type				0.329
GI cancer	8 (47.1)	5 (45.5)	3 (50)	
Lung cancer	6 (35.3)	3 (27.3)	3 (50)	
GB–biliary cancer	3 (17.6)	3 (27.3)	0 (0)	
Dexamethasone				
Dose (mg, per cycle) *	6.5 [4.7, 9.0]	6.7 [4.5, 9.0]	5.6 [4.7, 9.0]	0.648
Duration (days, per cycle) *	4.0 [3.0, 4.0]	4.0 [3.5, 4.0]	3.5 [3.0, 4.0]	0.264
HbA1c (%)	7.5 ± 1.7	7.9 ± 1.7	6.8 ± 1.4	0.216
FBS (mg/dL) *	141.0 [120.5, 165.5]	145.0 [131.0, 170.0]	126.0 [111.0, 142.0]	0.606
Duration of diabetes (years)	5.9 ± 6.9	8.4 ± 7.4	1.2 ± 1.4	**0.009**
Number of antihyperglycemic agents				0.193
Monotherapy	4 (23.5)	1 (9.1)	3 (50)	
Combination therapy	13 (76.5)	10 (90.9)	3 (50)	
- Dual combination	7 (41.2)	6 (54.5)	1 (16.7)	
- Triple combination	6 (35.4)	4 (36.4)	2 (33.3)	
Insulin use	2 (11.8)	1 (9.1)	1 (16.7)	1.000
AST (IU/L) *	19.0 [17.0, 33.0]	17.0 [15.5, 27.0]	32.0 [19.0, 34.0]	0.245
ALT (IU/L)	22.0 [13.0, 31.0]	17.0 [12.0, 31.5]	24.5 [18.3, 30.0]	0.725
eGFR (mL/min/1.73 m^2^)	89.2 ± 17.9	87.2 ± 17.0	93.6 ± 21.1	0.524

The data of normally distributed variables are presented as the mean ± SD. Skewed data * are expressed as the median (25 and 75% interquartile range). The frequencies and percentage relative frequencies were used for categorical variables. Bold number indicates statistically significant values. We have added an explanatory note in the table footer accordingly. ALT, alanine aminotransferase; AST, aspartate transaminase; BMI, body mass index; DXM, dexamethasone; eGFR, estimated glomerular filtration rate; FBS, fasting blood sugar; GB, gallbladder; GI, gastrointestinal; HbA1c, glycated hemoglobin; SD, standard deviation.

**Table 2:** Estimated mean glucose levels during first and second cycles of chemotherapy using linear spline models.

	First Cycle Estimate	*p*-Value	Second Cycle Estimate	*p*-Value	First to Second Cycle Difference	*p*-Value
First cycle (n = 17)						
(Intercept)						
~Knot 1	**4.41 (2.39 to 6.43)**	**<0.001**				
Knot 1~knot 2	**7.44 (4.36 to 10.52)**	**<0.001**				
Knot 2~knot 3	−0.23 (−0.48 to 0.02)	0.071				
Knot 3~	**−0.50 (−0.64 to −0.35)**	**<0.001**				
First and second cycles (n = 9)						
(Intercept)						
~Knot 1	2.61 (−0.21 to 5.43)	0.070	**4.71 (2.56 to 6.86)**	**<0.001**	−2.10 (−5.65 to 1.44)	0.245
Knot 1~knot 2	**9.26 (5.31 to 13.22)**	**<0.001**	0.82 (−2.91 to 4.56)	0.666	**8.44 (3.00 to 13.88)**	**0.002**
Knot 2~knot 3	0.09 (−0.23 to 0.41)	0.588	−0.19 (−0.52 to 0.14)	0.262	0.28 (−0.18 to 0.73)	0.237
Knot 3~	**−0.90 (−1.08 to −0.71)**	**<0.001**	**−0.96 (−1.20 to −0.71)**	**<0.001**	0.06 (−0.25 to 0.37)	0.713

Knot 1 was 1 h before dexamethasone treatment; knot 2 and knot 3 were 5.5 h and 55.5 h after dexamethasone treatment, respectively. Bold numbers indicate statistically significant values.

**Table 3:** Comparison of CGM parameters during first and second cycles of chemotherapy (n = 9).

	First Cycle	Second Cycle	*p*-Value
TIR (%)	33.11	45.22	**0.020**
TAR (%)	66.89	52.78	**0.003**
TBR (%)	0.11	2.06	0.305
GMI (%)	9.52	8.40	**0.020**
Mean glucose (mg/dL)	226.67	194.33	**0.022**
SD (mg/dL)	60.44	62.67	0.691

Bold numbers indicate statistically significant values. CGM, continuous glucose monitoring; GMI, glucose management indicator; TAR, time above range; TBR, time below range; TIR, time in range; SD, standard deviation.

## Data Availability

The data can be obtained from the corresponding author upon reasonable request.

## Supplementary Materials

The following supporting information can be downloaded at: https://www.mdpi.com/article/10.3390/biomedicines13051101/s1, Figure S1: Overview of study flow; Figure S2. A plot of linear spline model from the first and second cycles of dexamethasone-containing chemotherapy (n = 9); Table S1. Baseline characteristics of the 9 patients who received gliclazide; Table S2. Estimated mean glucose levels during the first cycle of chemotherapy using linear spline models (exploratory analysis).
