# Supplementary material for: Add-On Treatment with Gliclazide for Cancer Patients with Type 2 Diabetes Undergoing Cyclic Glucocorticoid-Containing Chemotherapy
Source: Biomedicines. 2025 May 1;13(5):1101. doi: 10.3390/biomedicines13051101 (PMC12109127; doi:10.3390/biomedicines13051101)
Supplement: Supplementary file 1 [file biomedicines-13-01101-s001.zip › biomedicines-3597159-supplementary.pdf]

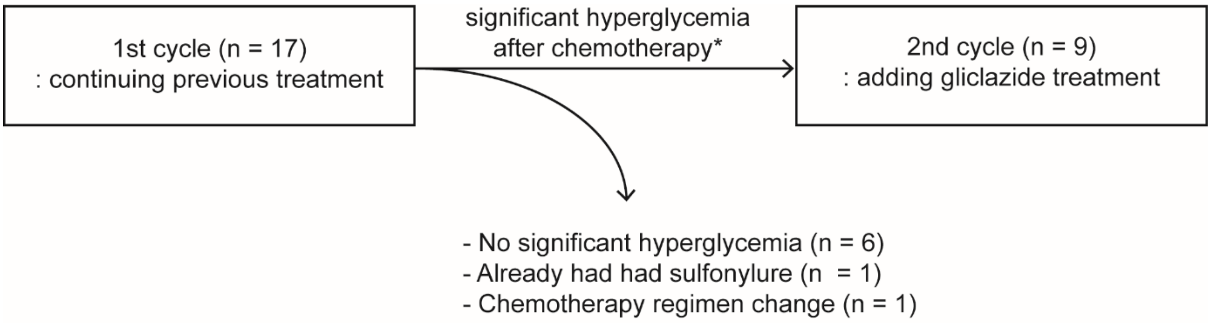

Supplementary Figure S1. Overview of study flow. *Significant hyperglycemia was defined as having a 20% or more increase in mean glucose value after the administration of dexamethasone compared to that on day 0.

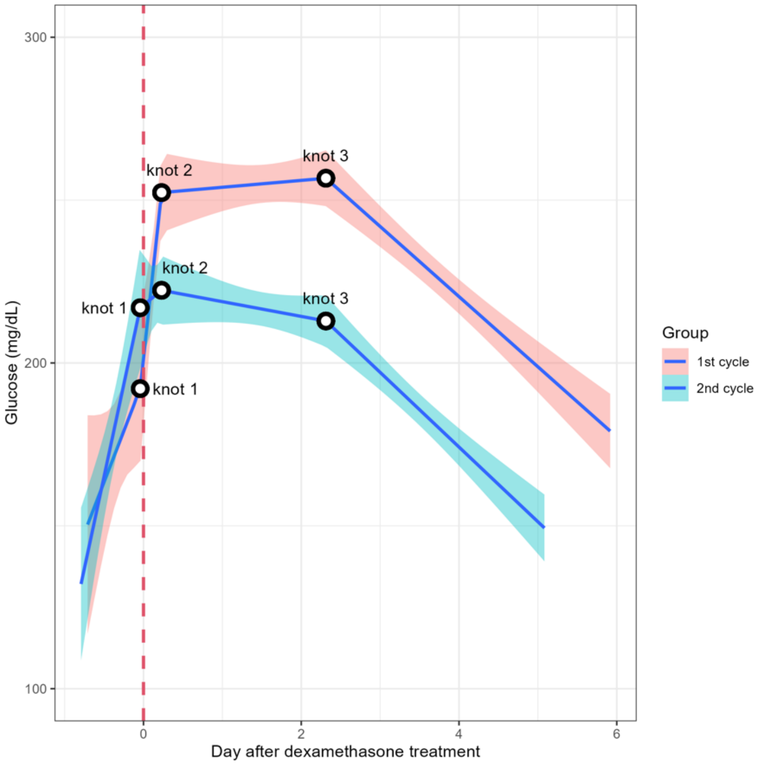

Supplementary Figure S2. A plot of linear spline model from the first and second cycles of dexamethasone-containing chemotherapy (n = 9). Three knots positioned at 1 h before dexamethasone treatment, and 5.5 h and 55.5 h after dexamethasone treatment**.**

Supplementary Table S1. Baseline characteristics of the 9 patients who received gliclazide

	**Patients (n = 9)**
Age	63.4 ± 10.1
Male (%)	6 (66.7)
Height (cm)	163.6 ± 7.7
Weight (kg)	64.5 ± 11.1
BMI (kg/m^2)	24.0 ± 3.0
Waist circumference (cm)	86.5 ± 5.6
GI cancer	4 (44.5)
Lung cancer	2 (22.2)
GB-biliary cancer	3 (33.3)
Dose (mg, per cycle)*	6.7 [4.5, 9]
Duration (days, per cycle)*	4 [3, 4]
HbA1c (%)	8.2 ± 1.6
FBS (mg/dL)*	155.5 [126.3, 187]
Duration of diabetes (years)	9.1 ± 7.8
Monotherapy	0 (0)
Combination therapy	9 (100)
- Dual combination	6 (66.7)
- Triple combination	3 (33.3)
Insulin use	1 (11.1)
AST (IU/L)*	17 [14, 27]
ALT (IU/L)	17 [11, 29]
eGFR (mL/min/1.73 m^2)	88.9 ± 18.6

Data of normally distributed variables are presented as the mean ± SD. Skewed data* are expressed as the median (25 and 75% interquartile range). The frequencies and percentage relative frequencies were used for categorical variables. ALT, alanine aminotransferase; AST, aspartate transaminase; BMI, body mass index; DXM, dexamethasone; eGFR, estimated glomerular filtration rate; FBS, fasting blood sugar; GB, gallbladder; GI, gastrointestinal; HbA1c, glycated hemoglobin; SD, standard deviation.

Supplementary Table S2. Estimated mean glucose levels during the first cycle of chemotherapy using linear spline models (exploratory analysis)

	First Cycle Estimate	*p*-Value
(Intercept)		
~Knot 1	**4.41 (2.40 to 6.43)**	**<0.001**
Knot 1~knot 2	**7.43 (4.47 to 10.39)**	**<0.001**
Knot 2~knot 3	**−0.26 (−0.42 to -0.11)**	**0.001**
Knot 3~	**−0.63 (−0.85 to −0.41)**	**<0.001**

Knot 1 was 1 h before dexamethasone treatment; knot 2 and knot 3 were 5.5 h and 80 h after dexamethasone treatment, respectively. Bold numbers indicate statistically significant values.
